# Ontogenetic and Experience-Dependent Changes in Defensive Behavior in Captive-Bred Hawaiian Bobtail Squid, *Euprymna scolopes*

**DOI:** 10.3389/fphys.2018.00299

**Published:** 2018-03-29

**Authors:** Kia Seehafer, Samantha Brophy, Sara R. Tom, Robyn J. Crook

**Affiliations:** ^1^Department of Biology, Sacramento State University, Sacramento, CA, United States; ^2^Department of Biology, San Francisco State University, San Francisco, CA, United States

**Keywords:** defensive behavior, *Euprymna*, habituation, learning, memory, squid

## Abstract

Cephalopod molluscs are known for their extensive behavioral repertoire and their impressive learning abilities. Their primary defensive behaviors, such as camouflage, have received detailed study, but knowledge is limited to intensive study of relatively few species. A considerable challenge facing cephalopod research is the need to establish new models that can be captive bred, are tractable for range of different experimental procedures, and that will address broad questions in biological research. The Hawaiian Bobtail Squid (*Euprymna scolopes*) is a small, tropical cephalopod that has a long history of research in the field of microbial symbiosis, but offers great promise as a novel behavioral and neurobiological model. It can be bred in the laboratory through multiple generations, one of the few species of cephalopod that can meet this requirement (which is incorporated in regulations such as EU directive 2010/63/EU). Additionally, laboratory culture makes *E. scolopes* an ideal model for studying ontogeny- and experience-dependent behaviors. In this study, we show that captive bred juvenile and adult *E. scolopes* produce robust, repeatable defensive behaviors when placed in an exposed environment and presented with a visual threat. Further, adult and juvenile squid employ different innate defensive behaviors when presented with a size-matched model predator. When a 10-min training procedure was repeated over three consecutive days, defensive behaviors habituated in juvenile squid for at least 5 days after training, but memory did not appear to persist for 14 days. In contrast, adult squid did not show any evidence of long-term habituation memory. Thus we conclude that this species produces a range of quantifiable, modifiable behaviors even in a laboratory environment where ecologically-relevant, complex behavioral sequences may not reliably occur. We suggest that the lack of long-term memory in adult squid may be related to their less escalated initial response to the mimic, and thus indicates less motivation to retain memory and not necessary inability to form memory. This is the first demonstration of age-related differences in defensive behaviors in *Euprymna*, and the first record of habituation in this experimentally tractable genus of squid.

## Introduction

Cephalopod molluscs have received intensive study of their behaviors and nervous systems, due to their vertebrate-like cognitive abilities, neurally-controlled skin pigmentation that enables rapid camouflage and signaling, and dynamic behavioral repertoires that are produced reliably in captive settings. Molluscan models, including cephalopods, have also been instrumental in advancing our understanding of the cellular mechanisms that underlie behavioral plasticity, from the simplest forms of learning (Glanzman, 2010) to more complex cognitive processes, including problem solving and observational learning (Fiorito and Scotto, 1992; Richter et al., 2016). Cephalopods' behaviors are readily modified by experience, including exposure to threats (Crook and Basil, 2008; Crook et al., 2009, 2011; Alupay et al., 2014; Oshima et al., 2016), but due to the difficulty of culturing most cephalopod species from eggs in laboratory settings, less is known about ontogenetic changes to behavior, particularly in squid.

The Hawaiian bobtail squid, *Euprymna scolopes* is a small, tropical cephalopod that has been well studied for its symbiotic relationship with the bioluminescent bacteria, *Vibrio fischeri* (Nyholm and McFall-Ngai, 2004; Lee et al., 2009). *E. scolopes* is one of the few cephalopod species that can be reared successfully through multiple generations in laboratory settings, and is experimentally tractable and relatively easy to keep. It is therefore a promising model for behavioral and neurobiological studies (Zepeda et al., 2017), but currently there is limited literature on its behavior.

The genus *Euprymna* contains at least six species, with cosmopolitan distribution throughout southeast Asia and Australasia and robust local population structures (Kimbell et al., 2002). Previous studies examining the defensive behaviors and personality traits of *Euprymna tasmanica* suggest that innate behavioral responses to threatening stimuli remain fairly constant across the squid's lifespan (Sinn, 2005; Sinn and Moltschaniwskyj, 2005; Sinn et al., 2008, 2010), but experience-dependent effects have not been tested. Wild observations of *E. scolopes* by divers suggest these squid employ a range of defensive behaviors, which may be combined or performed in dynamic sequences when animals were presented with persistent threats (Moynihan, 1983; Anderson and Mather, 1996). Here, for the first time, we examine ontogenetic and experience-dependent effects on defensive behaviors in captive bred *Euprymna scolopes*. By reducing environmental complexity and presenting a stereotyped threat stimulus over multiple trials, we show that juveniles are more reactive to threats than adults, and that juvenile responses can be habituated both within and across days of training. In contrast, adult squid displayed low-level responses to the presented threat, and did not show evidence of within- or across-day habituation under identical training conditions to juveniles.

## Methods

### Animals

First and second generation, captive bred squid originated from six wild-caught *E. scolopes*, collected in the waters surrounding O'ahu, Hawaii. Subjects were reared from birth in the laboratory, in continuously circulating artificial seawater maintained at a temperature of 24.5°C. Hatchling and juvenile squid were fed *ad libitum* on a combination of live mysid shrimp (*Amerimysis bahia*) and grass shrimp (*Palaemonetes*)spp. and adult squid were fed live grass shrimp twice per day. *E scolopes* is short lived, growing to sexual maturity at around 55–70 days post hatching, and entering senescence between 90 and 120 days (Lee et al., 2009). Juvenile squid used in this study were 30–37 days post hatching at the outset of experiments, and adults ranged in age from 62 to 90 days post hatching.

Squid were housed in groups of 4–6 in round enclosures 26 cm in diameter. About 2.5 cm of sand covered the mesh bottom of the enclosure and water level was maintained at ~24 cm, flowing in from an overhead pipe and out through the mesh bottom. At the end of experiments animals remained in the lab in the breeding colony, until they died of natural causes between 120 and 241 days post hatching.

In the United States, cephalopods are not included in federal laws governing the use and welfare of research animals, thus no protocol or approval number was required for this study. As such, all applicable international, national, and/or institutional guidelines for the care and use of animals were followed.

### Experimental apparatus

For behavioral tests, one squid was removed from its home enclosure and transferred to a round crystallization dish (“100 mm” size for juveniles and “125 mm” size for adults), filled with water taken from the home tank system but containing no internal structure or substrate. The test arena was enclosed in a white blind that prevented the squid from seeing the predator mimic before it was immersed in the trial arena. A camcorder (Sony, HDR-XR200) was positioned 70 cm directly above the arena, and all trials were filmed for later behavioral analysis. The entire experimental setup was enclosed inside a black curtained sub-room that visually isolated the squid from the experimenters and other stimuli.

**Figure 1:**
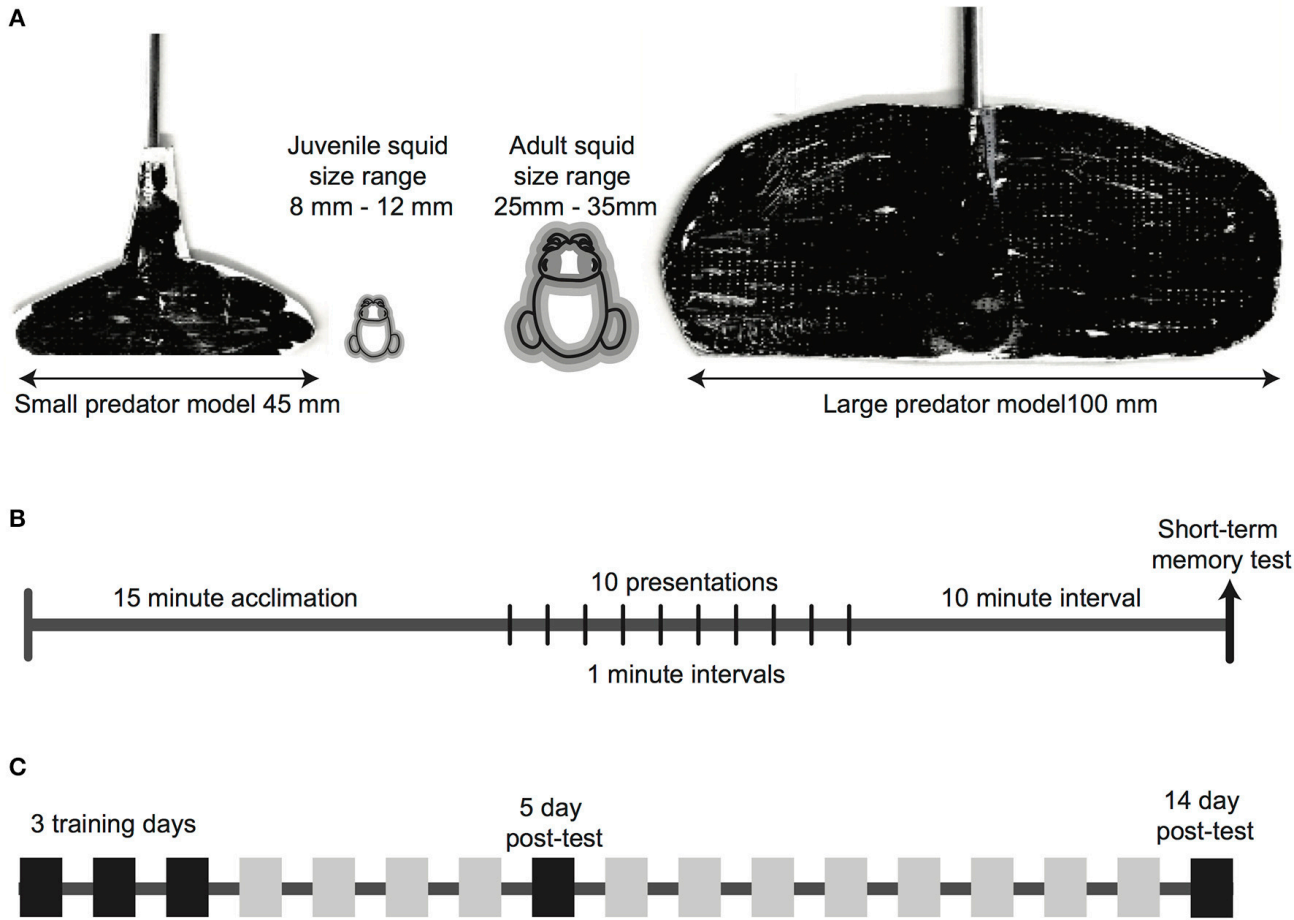
Methodological details of the experiment. **(A)** Predator mimics shown alongside a squid outline, showing the relative size of the subject and the model. Squid outlines show the average squid size (black line), with the gray zone showing the range of sizes in each age class. **(B)** Timeline of each experimental trial, which was repeated on each experiment day. A 15-min acclimation period was followed by 10 presentations of the mimic, with a 1-min inter-presentation interval. A single presentation, which functioned as a short-term memory test, was given 10 min after the conclusion of the training sequence. **(C)** Timeline of the full experiment. Trials occurred on three consecutive training days, and long-term memory was tested 5 days, and again at 14 days after the third training day.

We used two predator mimics, size matched to the two age classes of squid, with each ~4x the body length of the squid being tested (Figure 1A). We based the shape and size of the mimic on initial observations of squid not used in the subsequent experiments, that showed strong, innate defensive reactions to a looming visual stimulus that was longer than it was tall, and little to no response to a stimulus that was tall and narrow. We considered that these two orientations may have resembled benthic and pelagic fish predators, respectively. Mimics were constructed from white duct tape attached to a laboratory measuring spoon, cut to a size and shape that produced strong defensive reactions in initial testing. Mimics were colored black with permanent marker to contrast strongly with the white arena surround. A trained experimenter controlled the movement of the mimic. We chose to move mimics by hand instead of by an automated movement. Animals were unrestrained and moved freely about the arena, and it was crucial that the mimic was presented directly in front of the squid at each presentation, to accurately represent a looming predator. Although this undoubtedly introduced some variation into the speed and positioning of the mimic, we considered that this was preferable to an automated movement that may have collided with the moving squid, or traveled in a fixed line that did not necessarily approach the squid's position. Typical approach speeds were measured at ~2 cm/s.

### Procedure

Each experimental trial followed the same sequence (Figure 1B). Squid were given a 15-min acclimation period after being moved into the test arena, in which there was no visual or physical disturbance. Video recording commenced in the final minute of acclimation and continued throughout the trial. After the acclimation period ended, 10 presentations of the predator mimic were given, with a 1-min inter-presentation interval. Mimics were always introduced to the arena at the edge directly opposite the squid, moved across the arena until directly in front of the squid (within 10 mm of the arms), then removed, without making contact with the squid's body.

A single short-term memory test was given 10 min after the final presentation of the mimic. This test presentation was identical to all other presentations.

We repeated this procedure for three consecutive “training days” (Figure 1C), then tested for long-term memory retention 5 days after the final training day, and again at 14 days after the final training day, so the longest unreinforced interval tested was 9 days (between the 5 and 14th day). Experimental procedures on the two long-term test days were identical to the training days.

### Data analysis and statistical procedures

#### Behavioral coding

**Figure 2:**
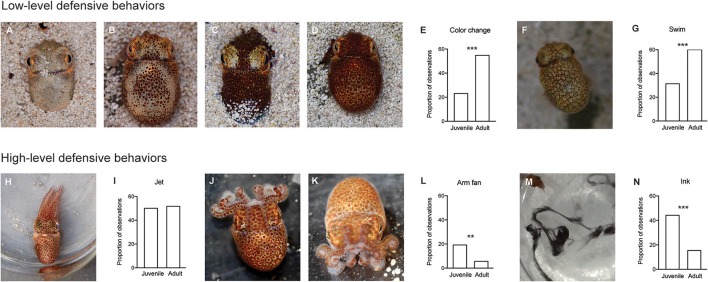
Behaviors produced in response to the predator mimic were classed as high- or low-level responses. On the first training day, adults were more likely to employ low-level responses, while juveniles were more likely to produce high-level responses. Top panel: **A–D** a color change was recorded when the animal changed from any one of four defined patterns to another. We grouped the continuous color change variable into four discrete patterns: **(A)** Pale, **(B)** Dice (see Moynihan, 1983), **(C)** Mottled or **(D)** Dark. Photographs show a squid on sandy substrate for illustrative purposes only. Trials were conducted with no substrate. **(E)** Color change was more likely to be employed by adult squid (*n* = 15) compared with juveniles (*n* = 12) (Chi-square test, *p* < 0.001). **(F)** Fin swimming, another low leve defensive behavior, was characterized by slow movement (<1 bodylength/s) extended, undulating fins, and rounded mantle. **(G)** Adults employed fin swimming more frequently than juveniles (*p* < 0.001). Bottom Panel: we recorded three high-level defensive behaviors. **(H)** Jetting was characterized by extended arms, rapid movement (>1 bodylength/second), folded fins and contracted mantle. **(I)** There was no difference in the frequency of jetting in response to the predator mimic between juvenile and adult squid. **(J,K)** A characteristic arm posture “Arm Fan” employed by *E.scolopes* in response to the predator mimic. **(J,K)** show two different angles. **(L)** Arm fan was produced more frequently by juveniles (*p* = 0.02). **(M)** Inking was the highest level defensive response we recorded. **(N)** Inking was more frequent in juveniles than in adults (*p* < 0.001).

We categorized defensive behaviors into six classes, and ranked them from lowest to highest level, based on previously published reports (Moynihan, 1983; Sinn and Moltschaniwskyj, 2005; Sinn et al., 2008, 2010). Ranks (Figure 2) were: no response (rank = 0; not shown on Figure 2), color change (rank = 1), avoidance swimming using the fins (rank = 2), escape jetting using the mantle (rank = 3), a distinctive arm posture we term “arm fan” (rank = 4), and inking (rank = 5). For each presentation, we recorded whether or not a squid performed any of these behaviors, and allocated a score to each response that corresponded to the most escalated behavior observed (for example, a squid that changed color, swam away and then produced an arm fan would receive an escalation score of 4). While squid often employed several behaviors in response to one presentation of the stimulus (as has been described previously by Anderson and Mather, 1996), we chose a simplified method of recording only the single most escalated response, as we hypothesized that this would be most likely to capture declines in responses due to habituation learning. We also measured the time the squid took after each presentation to return to a quiescent state, usually characterized by sitting on the base of the arena or hovering in place using fin swimming. Because squid occasionally stopped and then restarted responses to the stimulus, we considered quiescence to have occurred when the squid remained still for at least 5 s.

Behavioral data from video records was coded independently by at least two experimenters, and inter-rater reliability exceeded 90%. In addition, 4 “untrained” observers re-scored ~10% of all video files, and were given only written and still-image descriptions of the behaviors recorded by trained observers. Untrained scorers had observed *E. scolopes* behavior informally in the lab, but did not take part in experiments and were blind to expectations and previous data collection for the study. “Untrained” observers replicated the trained observers scores in 82% of the observations, a level we deemed acceptable. Where mismatched scores for either trained or untrained observers were recorded, the senior author (RJC) re-scored those observations, and acted as the “tie-breaker” in determining the final score for that presentation.

#### Statistical analyses

Behavioral responses were recorded for every presentation in the first training trial to compute response proportions (Figure 2). Statistical analyses were conducted in GraphPad Prism 6.0. We used a Chi-Square test with Yates' correction to compare frequencies between adults and juveniles for each behavior.

**Figure 3:**
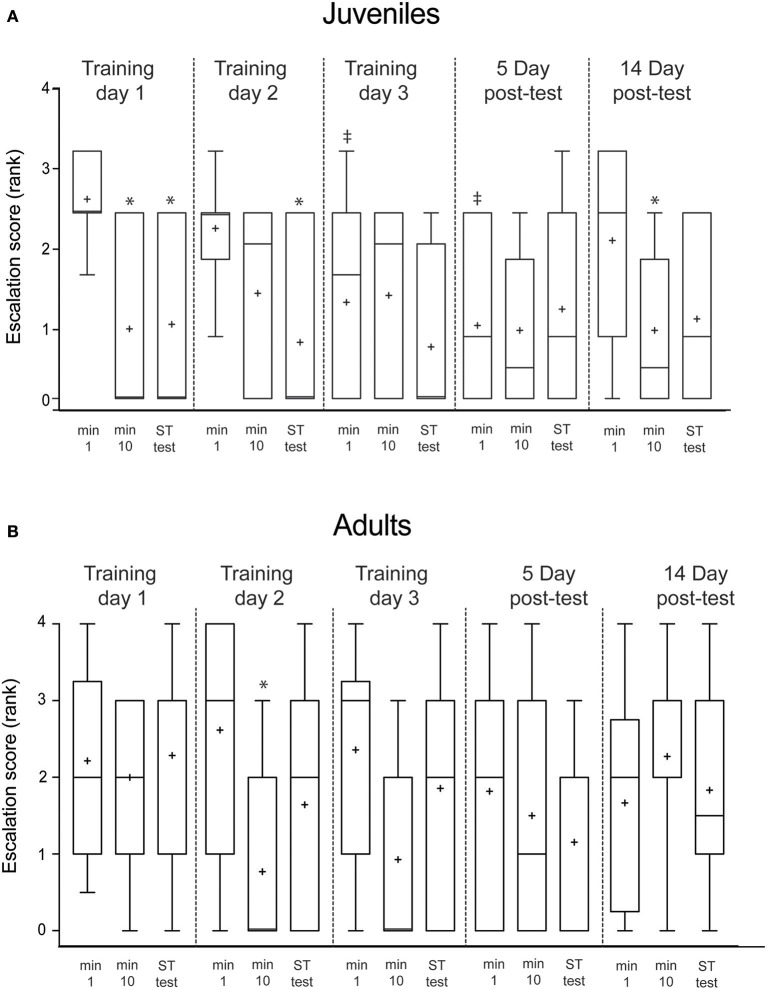
Escalation score (the highest ranked behavior produced in response to presentation of the predator mimic) declined in juveniles both within and across days, but did not decline in adults. Ranks were: no response = 0, color change = 1, avoidance swim = 2, jet = 3, arm fan = 4, ink = 5. **(A)** Mean escalation scores of juveniles (*n* = 12) plotted for the first minute, final (tenth) minute and short-term test (10 min after training) for each day of the experiment. **(B)** The same intervals plotted for adult squid (*n* = 15). Boxes show 25/75 percentiles, whiskers are 10–90th percentiles. Median is indicated with a line and mean with +. Comparisons are made with Wilcoxon signed-ranks tests. Comparisons within each day (min 1 vs. min 10 or vs. test, indicating short-term memory aquired within a trial) are denoted with ^*^. Across trial comparisons for the first minute of Day 1 vs. first minutes on subsequent days (indicating long-term memory) are denoted with ‡.

For ease of interpretation, on figures we show only behavioral responses for the first, tenth and test presentation for each day. Ranked escalation scores (Figure 3) were compared with Wilcoxon signed-ranks tests for comparisons made between the first and tenth minute of each test, and the first minute and test presentations. We also compared scores between the first minute on the first training day, and each of the first minutes on each subsequent day. Sequential Bonferroni corrections were applied to planned comparisons.

**Figure 4:**
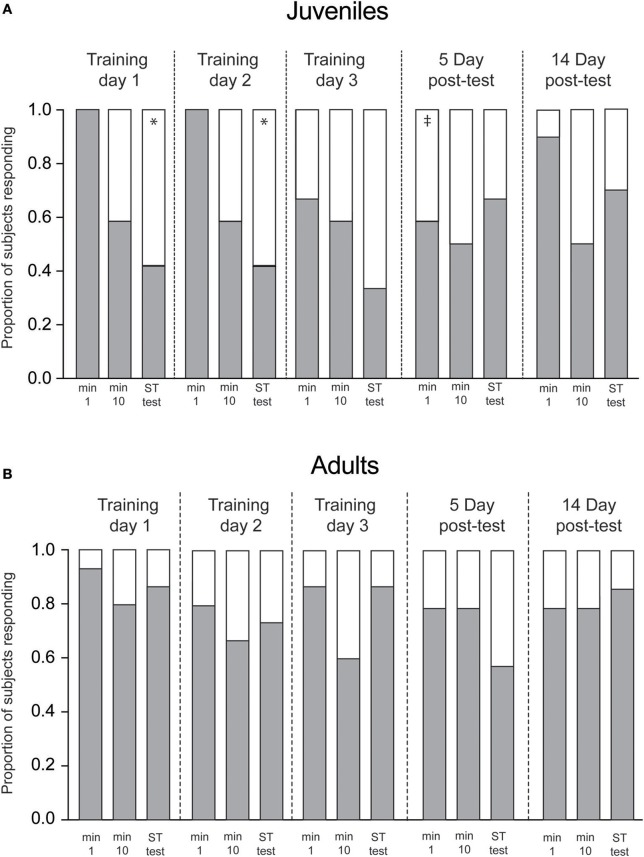
Proportion of response (gray) and non-response (white) to Presentation 1, 10 and short-term tests on each training day. **(A)** Juveniles (*n* = 12) showed significant declines in responses from the first presentation to the short-term memory test on Day 1 and Day 2. Long-term memory was present at the 5-day interval, but not the 14-day interval. **(B)** Adult squid showed no change in response/nonresponse rate over the training sequence. Bars show proportion of squid responding on each trial. Statistical comparisons made with Fisher's Exact Tests Comparisons within each day (min 1 vs. test, indicating short-term memory acquired within a trial) are denoted with ^*^. Significant across trial comparisons for the first minute of Day 1 vs. first minutes on subsequent days (indicating long-term memory) are denoted with ‡.

**Figure 5:**
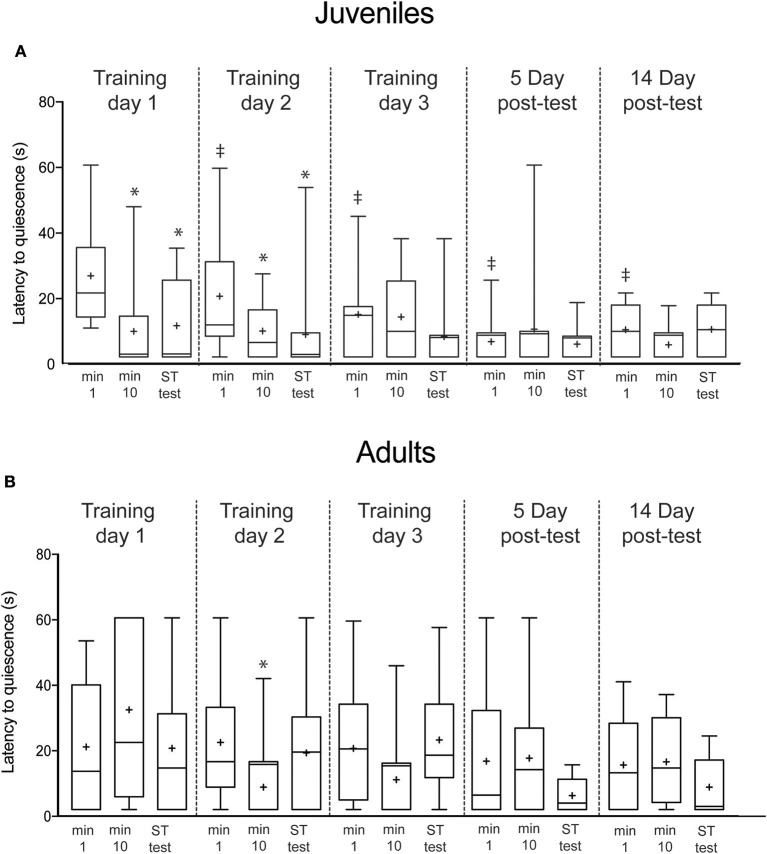
Latency to quiescence after the first, tenth and short-term test presentation of the predator mimic. **(A)** showed significant declines in responses from the first to last training trials on Day 1 and Day 2, and showed short-term retention on each day. Long-term memory appeared on Day 3 and was present at the 5-day interval, but not the 14-day interval. **(B)** Adult squid showed no sustained changes in response/nonresponse rate over the training sequence. Boxes show 25/75 percentiles, whiskers are 10–90th percentiles. Median is indicated with a line and mean with +. Statistical comparisons made with paired *t*-tests. Comparisons within each day (min 1 vs. min 10 or vs. test, indicating short-term memory acquired within a trial) are denoted with ^*^. Across trial comparisons for the first minute of Day 1 vs. first minutes on subsequent days (indicating long-term memory) are denoted with ‡.

To compare the proportions of “response vs. no response” (Figure 4), we used Fisher's Exact Tests. Latency to quiescence measures (Figure 5) conformed to the normal distribution (Bartlett's Test: adults: *p* = 0.23, juveniles; *p* = 0.13) and were analyzed with ANOVA followed by paired *t*-tests for within and across-trial comparisons. A *p* < 0.05 was considered significant.

## Results

### Frequencies of high- and low-level defensive behaviors vary with age

Innate responses to the predator model varied between juvenile and adult squid. On the first day of training, when squid were experiencing the predator mimic for the first time, adult squid were more likely to display low level defensive behaviors of color changes (χ^2^ = 34.9, *p* < 0.001) and fin swimming, (χ^2^ = 27.3, *p* < 0.001; See Figures 2A–G) compared with juveniles, while the high level defensive behaviors of arm postures and inking were more frequently employed by juveniles than by adults (arm fan: χ^2^ = 13.5, *p* = 0.002; ink χ^2^ = 34.2, *p* < 0.001; Figures 2J–N). Instances of Jetting did not differ between the two age classes (Figures 2H,I).

### Juvenile squid show consistent evidence of short- and long-term defensive habituation, but adults do not

In juvenile squid, the average escalation score (the rank value of the most highly escalated behavior produced at each presentation) declined significantly from the first to the tenth minute of training on the first training day (*p* = 0.03; Figure 3A), indicating rapid habituation to the predator mimic. Reduced responses persisted at the short-term memory test (*p* = 0.03). There was no long-term memory apparent after a single day of training, as the escalation score for the first presentation on the second day was not lower than that on the first day. Scores declined again over the course of the second day of training, with short-term memory apparent (*p* = 0.02). By the third training day, long term habituation memory was present, as the initial score was significantly lower than that of the first training day (*p* = 0.03) Escalation scores remained low at the first presentation of the 5-day post-test (0.02), indicating stable long-term memory. Escalation scores on day 14 were not significantly different than for Day 1, indicating no memory of the procedure persisted at this interval.

The results for adult squid differed in that there was no apparent habituation on Day 1 of training, and short-term memory first appeared on the second training day (*p* = 0.03; Figure 3B). Unlike in juvenile squid, long-term (>24 h) reduction in escalation scores was not apparent in adult squid.

We also measured learning by tracking the proportion of presentations to which squid made any measureable response, vs. no response at all (Figure 4). Similar to results described above, juvenile squid showed both within- and across-trial habituation to the predator mimic (Figure 4A), with positive responses declining over the first day of training (*p* = 0.02) and the second day of training (p = 0.016). Proportions of responses were intermediate for the third training day, but long-term memory was apparent at the 5-day post-test, where response rate was significantly lower than on the first trial of the first training day (*p* = 0.04). By the 14th day after training proportions were not different from Day 1, matching the results observed for escalation scores. Among adult squid, response rates were generally high across all trials, with no significant declines observed at any point in the experimental sequence.

Lastly, we recorded the latency for squid to return to quiescence after each presentation of the predator mimic. Among juvenile squid, latency to quiescence declined in the first trial (*t* = 4.9, *p* = 0.004; Figure 5A), and remained low for the short-term memory test (*t* = 3.1, *p* = 0.01). The same pattern was observed on Day 2 (min 1 vs. min 10, *t* = 2.2, *p* = 0.047; min 1 vs. ST memory test, *t* = 3.8, *p* = 0.003). By Day 3 long-term memory was apparent (min 1 Day 1 vs. min 1 Day 3, *t* = 2.1, *p* = 0.04). Latencies remained significantly reduced on both Day 5 (min 1 Day 1 vs. min 1 Day 5, *t* = 3.6, *p* = 0.0006) and Day 14 after training (min 1 Day 1 vs. min 1 Day 14, *y* = 2.8, *p* = 0.006).

Latency to quiescence for adults showed a somewhat different pattern to escalation and overall response rate. Although there was no learning apparent on Day 1 (Figure 5B), on the second training day a significant reduction in latency occurred by the tenth training trial (*t* = 2.3, *p* = 0.02), however, this short-term effect did not persist at the 10-min memory test, nor was there a similar significance pattern on Day 3. There was no evidence of across-trial reductions that would indicate long-term memory acquisition.

## Discussion

Here we show that *Euprymna scolopes* displays age-related changes to innate defensive behaviors, and also exhibits age-related differences in short-and long-term habituation. More broadly, we show that changes to the behaviors of these captive bred squid can be captured by relatively simple metrics, suggesting that this species is a promising behavioral and neurobiological model that warrants additional development and investigation.

Juvenile squid displayed escalated innate defensive behavior more frequently than adult squid, (where “innate” defensive behavior was defined as any behavior exhibited on the first day of training, given that squid were all captive bred and had no experience with real or simulated predators prior to experiments). Previous findings from a closely related species, *Euprymna tasmanica*, suggest similar ontogenetic shifts in defensive behavior, where juveniles exhibit strong defensive responses, sub adults a wide variety of responses, and adults solidify their responses as either “shy” or “bold” squid (Sinn and Moltschaniwskyj, 2005; Sinn et al., 2008). It is possible that since we tested different animals in the juvenile and adult age groups, the adults' physical (developmental) environment, which included frequent handling and constant presence of laboratory personnel, may have contributed to their less reactive defensive behavior, or that adult squid of this species are bolder in general. Although the juvenile squid in our study were sexually immature, in other respects their behavior and physiology appears largely indistinguishable from that of adults; their hunting behaviors and general diurnal patterns are identical to those of adults, and we observed no evidence of immaturity in their sensory or motor systems that might explain the differences in behaviors between adults and juveniles.

Escalation score and response/non-response data (Figures 3, 4) indicate that juveniles habituate in the short-term over each trial, and exhibit signs of long-term memory at the 5-day retention interval. Although it is possible that the within-trial declines we observed might be due to fatigue or some other non-mnemonic factor, such effects cannot account for the across-trial effect of reduced responses on the first presentation on subsequent days. Both within- and across-trial habitation have been demonstrated in other cephalopods (Angermeier and Dassler, 1992; Kuba et al., 2006a,b), and in a large number of other invertebrate species (see Byrne and Hawkins, 2015). We have recently demonstrated stable long-term associative memory in the species (Zepeda et al., 2017) but to our knowledge this is the first demonstration of long-term, non-associative learning in sepiolid squid.

While adult squid also showed some inconsistent signs of short-term habituation to the predator model, they never exhibited long-term retention. We propose several explanations for why adults failed to commit the predator mimic to long-term memory. First, it is possible that *E. scolopes* becomes less cognitively capable of consolidating long-term memory as it ages. Studies of cuttlefish (*S. officinalis*), show that two year old (geriatric) animals were slower to learn an avoidance task than one year old cuttlefish (Mather, 2008), and that in senescent cuttlefish, initial neural decline was motor and not sensory (Mather, 2008). This suggests that older or senescent cephalopods may have a failure to react to stimuli, even though they register it.

Second, it is possible that the predator mimic we used mimicked a predator specifically of juvenile squid. It is likely that wild *E. scolopes* face different predators at different size classes, but there is little available literature on predators of adult *E. scolopes*. Other studies on cephalopods have shown that familiarization with specific prey throughout early time periods leads to specific prey preferences (Darmaillacq et al., 2006a,b), which could be true also for predator recognition. It is possible that adult squid are not predated by benthic predators in the wild, and thus our mimic was less ecologically relevant to adults than to juveniles. Predation risk is generally related to body size, and as individuals grow they face different predators (Werner and Gilliam, 1984) Additionally, the adult *E. scolopes* in this study had no prior experience of predators; perhaps the lack of exposure during an early “sensitive period” in which predator recognition is solidified, contributed to the minimal responses we observed here.

Third, it is possible that our study was unable to clearly identify adult defensive behaviors. For example, adults may engage in burying as a defensive behavior more often than highly escalated and highly visible behavior such as inking; since our experimental set-up did not include substrate, we may have been unable to clearly distinguish burying behavior in adults from avoidant swimming or jetting.

## Conclusion

Captive bred *Euprymna* express ecologically relevant, easily quantifiable defensive behaviors in controlled experimental settings. *E. scolopes* exhibits age-dependent, innate defensive behaviors in response to a simulated predator threat test, and age-dependent differences in patterns of learning and memory. We propose that our study provides several important advances for the field of cephalopod research, which is currently challenged to meet higher ethical standards than in the past (see EU directive 2010/63/EU), to broaden its scope to improve the diversity of species typically used in studies, and to produce novel findings that enlighten broad questions in biological research. Here, we demonstrate the feasibility of using first and second generation captive bred specimens of *E. scolopes* in behavioral research, a more ethical alternative than using wild-caught specimens. Further, we show that squid, which are rarely used in behavioral experiments, express robust non-associative learning and memory, a finding that broadens knowledge of the diversity of behaviors in cephalopods. Finally, we suggest that studies such as those described here have the possibility of enlightening broad debates about origins of intelligence, the neuroanatomical and physiological underpinnings of neural plasticity, and the evolution of complex behaviors.

## Author contributions

KS and RC designed experiments; KS, SB, and ST conducted experiments; all authors analyzed data and conducted statistical analysis, all authors wrote the paper.

### Conflict of interest statement

The authors declare that the research was conducted in the absence of any commercial or financial relationships that could be construed as a potential conflict of interest.
